# Editorial for the Special Issue on the Progress of Emerging Hardware Development for Post-Moore’s Computing

**DOI:** 10.3390/mi14010193

**Published:** 2023-01-12

**Authors:** Yao-Feng Chang

**Affiliations:** Intel Corporation, Hillsboro, OR 97124, USA; yfchang@utexas.edu

The potential of machine learning and novel computing architecture can be exploited in the immediate future if more efficient hardware is developed that meets the special requirements of bio-inspired computing or unconventional computing schemes. In this area, non-volatile memory (NVM) technology using memristive devices (not restrained to any type of devices) and other emerging hardware systems in combination with neuromorphic systems and memcomputing (memristor + computing) offer a promising way to achieve such a milestone. As we were preparing this Special Issue, we had the following questions in mind: what is the future of post-Moore computing? How will the conventional computing system (e.g., Von Neumann architecture) be advanced in this new era? What is the current state of the art for post-Moore computing? In this Special Issue, we focus on the latest advancements, current challenges, and new opportunities in the world of emerging hardware development for post-Moore computing. Fundamentals and applications are both covered in this issue (a total of nine original papers). The fundamentals include novel materials, manufacturing techniques, and characterization, among others. In terms of applications, recurrent neural network [1], emerging hardware solutions and development for post-Moore computing (especially on ReRAM or memristive devices) [2,3,4,5], NVM device simulator [6], in-memory computing [7], physical unclonable function (PUF)- based secure authentication using spin-transfer torque magnetic random-access memory (STT-MRAM) [8], and 5G mobile communication [9] were discussed. The goal of this Special Issue is to stimulate the community by addressing the key issues on the topic in the hope that emerging hardware development for post-Moore computing will make a greater impact in our society. 

Several fabrication optimization and emerging device characterizations have been reported in this issue for the development of post-Moore computing hardware. For example, sneak path current (SPC) is the inevitable issue in crossbar memory array while implementing high-density storage/computing configuration. These crosstalks are attracting increasing attention, and the read accuracy in the crossbar architecture is deteriorated by the SPC. In that work, the sneak path current problem is observed and investigated by the electrical experimental measurements in the crossbar array structure with the half-read scheme. The read margin of the selected cell is improved by the bilayer-stacked structure, and the sneak path current is reduced ~20% in the bilayer structure. The voltage-read stress-induced read margin degradation has also been investigated, and less voltage stress degradation is showed in bilayer structure due to the intrinsic nonlinearity. The oxide-based bilayer-stacked resistive random-access memory (ReRAM) is presented to offer immunity toward sneak path currents in high-density memory integrations when implementing the future high-density storage and in-memory computing applications [2]. 

In addition to the many fabrication techniques reported here [3,4,5], this issue also covers a broad range of applications for post-Moore computing. Physical unclonable function (PUF), a hardware-efficient approach, has drawn a lot of attention in the security research community for exploiting the inevitable manufacturing variability of integrated circuits (IC) as the unique fingerprint of each IC. However, analog PUF is not robust and resistant to environmental conditions. In this Special Issue, we included an article focusing on the proposed digital PUF-based secure authentication model using the emergent spin-transfer torque magnetic random-access memory (STT-MRAM) PUF (called STT-DPSA for short). STT-DPSA is an original secure identity authentication architecture for Internet of Things (IoT) devices to devise a computationally lightweight authentication architecture which is not susceptible to environmental conditions. It provided a potential solution for IoT development as well as pathfinding for edge computing hardware development in the future [8]. 

We hope that this Special Issue on the progress of emerging hardware development for post-Moore computing will offer readers a comprehensive overview of the current state of the art in this fast-growing area of research, as well as an introduction to some of the newest techniques being developed in the field.
